# How many steps/day are enough? for children and adolescents

**DOI:** 10.1186/1479-5868-8-78

**Published:** 2011-07-28

**Authors:** Catrine Tudor-Locke, Cora L Craig, Michael W Beets, Sarahjane Belton, Greet M Cardon, Scott Duncan, Yoshiro Hatano, David R Lubans, Timothy S Olds, Anders Raustorp, David A Rowe, John C Spence, Shigeho Tanaka, Steven N Blair

**Affiliations:** 1 Walking Behavior Laboratory, Pennington Biomedical Research Center, Baton Rouge, LA, USA; 2 Canadian Fitness and Lifestyle Research Institute, Ottawa, ON, Canada; 3 School of Public Health, University of Sydney, Sydney, Australia; 4 Department of Exercise Science, Arnold School of Public Health, University of South Carolina, Columbia, SC, USA; 5 School of Health and Human Performance, Dublin City University, Dublin, Ireland; 6 Department of Movement and Sports Sciences, Ghent University, Ghent, Belgium; 7 Centre for Physical Activity and Nutrition, Auckland University of Technology; 8 Tokyo Gakugei University, Tokyo, Japan; 9 School of Education, University of Newcastle, New South Wales, Australia; 10 Health and Use of Time (HUT) Group, Sansom Institute for Heath Research, University of South Australia, Adelaide, Australia; 11 School of Sport Sciences, Linneaus University, Kalmar, Sweden; 12 Department of Neurobiology, Care Sciences and Society, Karolinska Institute, Sweden; 13 School of Psychological Sciences and Health, University of Strathclyde, Glasgow, UK; 14 Sedentary Living Lab, Faculty of Physical Education and Recreation, University of Alberta, Alberta, Canada; 15 Health Promotion and Exercise Program, National Institute of Health and Nutrition, Tokyo, Japan; 16 Departments of Exercise Science and Epidemiology & Biostatistics, Arnold School of Public Health, University of South Carolina, Columbia, USA

## Abstract

Worldwide, public health physical activity guidelines include special emphasis on populations of children (typically 6-11 years) and adolescents (typically 12-19 years). Existing guidelines are commonly expressed in terms of frequency, time, and intensity of behaviour. However, the simple step output from both accelerometers and pedometers is gaining increased credibility in research and practice as a reasonable approximation of daily ambulatory physical activity volume. Therefore, the purpose of this article is to review existing child and adolescent objectively monitored step-defined physical activity literature to provide researchers, practitioners, and lay people who use accelerometers and pedometers with evidence-based translations of these public health guidelines in terms of steps/day. In terms of normative data (i.e., expected values), the updated international literature indicates that we can expect 1) among children, boys to average 12,000 to 16,000 steps/day and girls to average 10,000 to 13,000 steps/day; and, 2) adolescents to steadily decrease steps/day until approximately 8,000-9,000 steps/day are observed in 18-year olds. Controlled studies of cadence show that continuous MVPA walking produces 3,300-3,500 steps in 30 minutes or 6,600-7,000 steps in 60 minutes in 10-15 year olds. Limited evidence suggests that a total daily physical activity volume of 10,000-14,000 steps/day is associated with 60-100 minutes of MVPA in preschool children (approximately 4-6 years of age). Across studies, 60 minutes of MVPA in primary/elementary school children appears to be achieved, on average, within a total volume of 13,000 to 15,000 steps/day in boys and 11,000 to 12,000 steps/day in girls. For adolescents (both boys and girls), 10,000 to 11,700 may be associated with 60 minutes of MVPA. Translations of time- and intensity-based guidelines may be higher than existing normative data (e.g., in adolescents) and therefore will be more difficult to achieve (but not impossible nor contraindicated). Recommendations are preliminary and further research is needed to confirm and extend values for measured cadences, associated speeds, and MET values in young people; continue to accumulate normative data (expected values) for both steps/day and MVPA across ages and populations; and, conduct longitudinal and intervention studies in children and adolescents required to inform the shape of step-defined physical activity dose-response curves associated with various health parameters.

## Background

The profound and robust benefits of a physically active lifestyle are recognized even for young people. Hence, worldwide, public health physical activity guidelines include special emphasis on children (typically 6-11 years) and adolescents (typically 12-19 years) [1-3], and there is growing interest in providing guidelines for preschool children [4]. Existing guidelines are commonly expressed in terms of frequency, time, and intensity of behaviour. However, with the technological advancement of objective monitoring of physical activity using pedometers and accelerometers, the opportunity now exists to offer another type of message that is congruent with these established guidelines. Although accelerometers offer a greater potential to study complex patterns of physical activity and sedentary behaviours in the course of research, the simple step output from both accelerometers and pedometers is gaining increased credibility in both research and practice as a reasonable approximation of daily ambulatory physical activity volume [5,6]. Of the two types of instrumentation, pedometers are more likely to be adopted for clinical and public health applications, and ultimately are also more likely to be embraced by the public themselves, due primarily to interpretability and relative low cost. Such users (i.e., clinicians, public health practitioners, and the general public) require good reference data and recommendations that are grounded in evidence in order to facilitate an effective step-based translation of public health guidelines. The purpose of this manuscript is to convey findings that inform a translation of public health guidelines for children and adolescents in terms of steps/day.

## Methods

This literature review was commissioned by the Public Health Agency of Canada (PHAC) and includes: 1) normative data (i.e., expected values); 2) incremental changes expected from interventions; 3) controlled studies translating cadence (i.e., steps/minute) to activity intensity; 4) studies of steps/day associated with time in moderate-to-vigorous physical activity (MVPA) under free-living conditions; and, 5) health outcome-related analyses (e.g., steps/day associated with valued health outcomes). In February 2010 a professional librarian executed the search strategy of CINAHL, ERIC, MEDLINE, PsycINFO, SocINDEX, and SPORTDiscus using the keywords (pedomet* or acceleromet*) and step* and ((physical activity) or walk*), limited to English language, and published since 2000 (an earlier review covered studies published before 2000 [7]). Articles were assembled, additional research was identified by reviewing article reference sections, and relevant content was abstracted and summarized by the first author. Where recent review articles were identified (e.g., normative data, interventions), the summarized results were presented to avoid redundancy and notable original articles selected to make specific points. Subsequently, researchers with practical experience collecting step data world-wide were invited to critically review the report, identify any additional relevant literature (including known articles in press), and intellectually contribute to this consensus document focused on children and adolescents. Study details were tabulated as appropriate. Any seeming inconsistencies in details catalogued within tables (e.g., instrument brand, model, numbers of decimal points, etc.) reflect underlying reporting inconsistencies as taken directly from original articles. The adult [8] and older adult/special populations [9] literature is reviewed separately.

## Results

### Normative data (expected values)

Normative steps/day data (or expected values) provide an indication of central tendency and variability and are useful for comparison purposes and interpreting change. However, they should not imply what children or adolescents "should" be taking, an index more appropriately described as a cut point or threshold value. Early work [7] that attempted to collate normative data (from studies published between 1980 and 2000) reported, based on a single study [10] published at the time, that we can expect 8-10 year old children to take 12,000 to 16,000 steps/day (lower for girls than boys). No data were available at the time to inform the number of steps/day that adolescents take. Since then, however, studies of young people's step data collected using pedometers and accelerometers have proliferated. In particular, two reviews have published normative data for children, together covering each sex-age group from 5-19 years of age [11,12]. Among children, boys average 12,000 to 16,000 steps/day and girls average 10,000 to 13,000 steps/day [11]. Although there are exceptions among countries, in general, peak values of mean steps/day occur before 12 years of age and decrease through adolescence until mean values of approximately 8,000 and 9,000 steps/day are observed in 18-year olds [12]. Across studies, physical education class participation generally contributes ≅9-24% of daily steps in boys and ≅11.4-17.2% in girls, and afterschool activity accounts for ≅47-56% and ≅47-59% (boys and girls respectively) of daily steps on school days [11]. Differences among countries are apparent, with children from North America (Canada and United States) showing lower values compared to other regions of the world, for example, when compared to European countries (Sweden, United Kingdom, Belgium, Czech Republic, France, Greece, and Switzerland), but especially when compared to Western Pacific countries (Australia and New Zealand) [12].

Beyond these reviews, a few specific references relative to normative data in young people are noteworthy. Vincent and Pangrazi [13] reported normative data for a U.S. sample in 2002 and at that time suggested that the mean values of 13,000 for U.S boys and 11,000 for U.S. girls could be used as reasonable standards for evaluation purposes. The U.S. President's Challenge: Physical Activity and Fitness Awards Program [14] adopted these same values to recognize physically active U.S. children (ages 6-17 years). A number of researchers from around the world have used these same values as cut points to evaluate data [15-17] although they can only be traced back to mean values based on a single descriptive study [13] of weekday step values obtained by 711 children aged 6-12 years living in the Southwestern U.S. The National Health and Nutrition Examination Survey (NHANES) in the U.S. adopted an accelerometer to objectively monitor physical activity in the 2003-2004 and 2005-2006 cycles; step data for children and adolescents collected in 2005-2006 have been recently published [5]. Once adjusted (i.e., reduced to those steps taken above a specified intensity) to make these accelerometer-determined step data interpretable against common pedometer-based scales, the results indicate that American young people aged 6-19 years take approximately 9,500 (boys) and 7,900 (girls) steps/day [5]. The 2005-2007 Canadian Physical Activity Levels Among Youth (CANPLAY) pedometer-determined physical activity data (based on a nationally representative sample of > 11,500 Canadian young people) are also just recently available [6,18]. The results indicate that Canadian young people aged 5-19 years of age take 12,000 (boys) and 11,000 (girls) steps/day [6]. To put these American and Canadian values in context, Amish young people 6-18 years of age, who purposefully refrain from adopting most technologies of modern living, average over 15,000 steps/day [19].

Tudor-Locke and Bassett [20] established pedometer-determined physical activity cut points for healthy adults: 1) < 5,000 steps/day (sedentary); 2) 5,000-7,499 steps/day (low active); 3) 7,500-9,999 steps/day (somewhat active); 4) ≥ 10,000-12,499 steps/day (active); and 5) ≥12,500 steps/day (highly active). These categories were reinforced in an updated review in 2008 [21] and in 2009 the original sedentary level was segmented into two additional levels: < 2,500 steps/day (basal activity) and 2,500 to 4,999 steps/day (limited activity) [22]. A similar (but sex-specific) graduated step index has been introduced for children (ages 6-12 years) [21]. Values for boys are: 1) < 10,000; 2) 10,000-12,499; 3) 12,500-14,999; 4) 15,000 - 17,499; and, 5) ≥ 17,500 steps/day. The corresponding values for girls are: 1) < 7,000; 2) 7,000-9,499; 3) 9,500-11,999; 4) 12,000 - 14,499 and, 5) ≥ 14,500 steps/day. The primary anchors for both of these sex-specific indices were based on a BMI-referenced criterion study of U.S., Australian, and Swedish children 6-12 years of age [23], and the appropriateness and generalizability of these cut points have been questioned [24]. The increments in the children's graduated step index were selected to be congruent with the adult index. For both sexes, each escalating category can be interpreted as "sedentary", "low active," "somewhat active," "active," and "highly active" similar to the labels used to define levels in the adult graduated step index, however, they have also been given labels of "copper," "bronze," "silver," "gold", and "platinum," in keeping with a style reflective of current physical activity and fitness award programs in the U.S. [14]. Another strategy might be to adopt existing graduated Canadian Physical Activity, Fitness and Lifestyle Approach (CPAFLA) [25] labels: Needs Improvement, Fair, Good, Very Good, and Excellent. It may be difficult to avoid unintentional potential for stigmatization using any qualitative label, however [21]. Only a single study has used this index to describe distribution of child data at this time [5] and we know of no validation study with regards to any other health parameter. An additional criticism of this version of children's graduated index could be that there are not enough "rungs on the ladder" leading up to the identified floor values separating 'sedentary' from 'low active." As indicated above, two additional levels have been added to the adult version. There is very little step data to inform an adolescent-specific graduated step index at this time.

Seventeen studies were identified that have reported relative achievement of various step-defined cut points and these are presented in Table 1 by publication year. Three of these have used the Vincent and Pangrazi [13] and/or President's Active Lifestyle Award [14] values of 13,000 for boys and 11,000 for girls (based on normative values for American adolescents [13]). Six have used BMI-referenced values (15,000 for boys, 12,000 for girls) described above [23]. Four have examined both of these, one used the Rowlands and Eston [16] cut points of 13,000 (boys) and 12,000 (girls) based on accumulating > 60 minutes in accelerometer-determined MVPA within the course of daily activity, one used the sex-specific children's graduated step index [23], and the remainder have used other variations. In general, 1) relatively more children than adolescents achieve a given cut point, 2) relatively more children and adolescents are able to achieve lower (rather than higher) cut points, and 3) relatively fewer U.S. children and adolescents achieve the same cut points when compared to those from other countries. Not included in the table is a study by Beets et al. [24] which evaluated the BMI-referenced cut points (e.g., in terms of sensitivity and specificity) but did not report the actual percentage of the sample achieving them.

**Table 1:** Studies reporting percent meeting select step-defined cut points in young people

Reference	Sample Characteristics	Instrument	Monitoring Frame	Cut points Used	% Meeting Specified Cut point		
Raustorp [59] 2003 Sweden	446 boys, 457 girls; school children; 6-14 years	Yamax Digiwalker SW-200 (Tokyo, Japan)	4 weekdays	VP/PALA	83% boys, 82% girls		

Cardon [15] 2004 Belgium	51 boys, 41 girls; elementary school children; 6.5-12.7 years	Yamax Digiwalker SW-200, Yamax Corp, Japan	7 days	VP/PALA BMI-referenced cut points	77% overall 63% overall		

Rowlands [16] 2005 UK	13 boys, 13 girls; primary school children; 8 to 10 years	Yamax Digi-Walker DW-200, Yamasa, Tokyo, Japan	6 days including 1 weekend day	VP/PALA BMI-referenced cut points	62% boys, 69% girls 38% boys, 54% girls		

Parfitt [60] 2005 UK	35 boys, 25 girls; primary school children; 9.8 to 11.4 years	Yamax Digiwalker SW-200, Yamasa, Tokyo, Japan	7 days	Rowlands and Eston 13000, 12000	25% boys, 30% girls		

Zizzi [61] 2006 USA	56 boys, 109 girls; high school students; 14 to 17 years	Accusplit Eagle 170	7 days	VP/PALA	< 25% overall		

Raustorp [62] 2007 Sweden	183 boys in 2000 85 boys in 2006 153 girls in 2000 83 girls in 2006; School children; 7 to 9 years	Yamax SW-200 Tokyo, Japan	4 weekdays	BMI-referenced cut points	2000: 67% boys, 90% girls 2006: 60% boys, 75% girls		

Duncan [63] 2007 UK	101 boys, 107 girls; primary school children; 8 to 11 years	New Lifestyles, NL2000, Montana, USA	4 days including 2 weekend days	BMI-referenced cut points	28.7% boys, 46.7% girls 41.2% of normal weight, 36.4% of overweight, 12.5% of obese		

Eisenmann [57] 2007 USA	269 boys, 339 girls; Midwestern elementary school children; 9.6 years	Digiwalker 200 SW	7 days	VP/PALA	not reported		

				BMI-referenced cut points	not reported		

				< 10000	19.3% boys, 33.9% girls		

				10000-12000	24.2% boys, 32.4% girls		

				12000-14000	24.2% boys, 22.7% girls		

				> 14000	32.3% boys, 10.9% girls		

Raustorp [64] 2007 Sweden	46 boys, 51 girls; School children; 12-14 years	Yamax SW-200	4 weekdays	BMI-referenced cut points	58% overall		

Reed [65] USA 2007	140 boys, 158 girls; elementary school children; 6 to 10 years	New Lifestyles Digi-Walker, SW-401, Yamax, Inc.	7 days	VP/PALA	41% overall		

					Grade	Boys	Girls

					First	35.70%	17.20%


					Second	55.00%	18.20%

					Third	78.60%	44.80%

					Fourth	48.10%	50.00%

					Fifth	51.40%	12.90%

Downs [66] Canada 2008	80 boys, 98 girls; Cree elementary school children; 9 to 11 years	Yamax SW-200 Digiwalker, Yamasa Corp., Tokyo, Japan	3 weekdays	BMI-referenced cut points	59% overall, 51% with central adiposity, 68% without		

Hohepa [67] 2008 New Zealand	95 boys, 141 girls; high school students; 12 to 18 years	NL-2000 New-Lifestyles Inc.	7 days	10,000	11.4% never met 24.4% sometimes met 49.7% often met 14.5% always met		

Laurson [55] 2008 USA	358 boys, 454 girls; elementary school children; 6 to 12 years	Digiwalker 200-SW	7 days	VP/PALA	41.3% boys, 45.6% girls		

				BMI-referenced cut points	23.2% boys, 31.5% girls		

				adult 10,000	80.2% boys, 63.2% girls		

				11,500 and 10,000 (boys, girls)	62.6% boys, 63.2% girls		

				10,000 and 11,000	80.2% boys, 45.6% girls		

Lubans [17] 2008 Australia	50 boys, 65 girls; adolescents recruited through schools; 14.15 ± 0.76 years	Yamax SW701	5 days including 1 weekend day	VP/PALA BMI-referenced cut points	49% boys, 52% girls 30% boys and girls		

Belton [68] 2009 Ireland	153 boys, 148 girls; primary school children; 6 to 9 years	Yamax Digwalker SW200	7 days	BMI-referenced cut points	62.2% boys, 74.7% girls		

Craig [6] 2010 Canada	5863 boys, 5639 girls; nationally representative sample 5-19 years	Yamax SW-200 (Tokyo, Japan)	7 days	BMI-referenced cut points 15,000 step/day 16,500 steps/day	23.2% male, 33.8% girls 23.2 male, 11.7% girls 13.8% male, 6.1% girls		

Tudor-Locke [5] 2010 USA	1281 boys, 1329 girls, nationally representative sample; 6-19 years	ActiGraph AM-7164, ActiGraph, Ft. Walton Beach, Florida (data treated to approximate pedometer output)	7 days	Sex-specific Children's Graduated Step Index (only in 6-11 year olds)	41.8% boys sedentary 21.2% girls sedentary (other categories presented in figures)		

Vincent and Pangrazi U.S. normative data [13] /President's Active Lifestyle Award (VP/PALA) [14]: 13,000 steps/day (boys) and 11000 steps/day (girls). BMI-referenced cut points [23]: 15,000 steps/day (boys) and 12,000 steps/day (girls). Sex-specific Children's Graduated Step Index for children (ages 6-12 years) [21]. Boys: 1) < 10,000; 2) 10,000-12,499; 3) 12,500-14,000; 4) 15,000 - 17,499; and, 5) ≥ 17,500 steps/day. Girls: 1) < 7,000; 2) 7,000-9,499; 3) 9,500-11,999; 4) 12,000 - 14,999 and, 5) ≥ 14,500 steps/day. For both sexes, each escalating category is respectively labelled "sedentary," "low active," "somewhat active," "active," and "highly active."

In summary, the updated normative data (i.e., expected values) based on international studies indicates that we can expect 1) among children, boys to average 12,000 to 16,000 steps/day and girls to average 10,000 to 13,000 steps/day; and, 2) steps/day values in adolescents to steadily decrease until approximately 8,000-9,000 steps/day are observed in 18-year olds.

### Interventions

A systematic review of studies that have used pedometers to promote physical activity in children and adolescents has been recently published [26]. Only 14 studies were identified, and 12 of these documented increases in physical activity. The magnitude of the intervention effects was variable and could very well reflect differences in study participants (e.g., children vs. adolescents, obese vs. non-obese), program factors, study design (e.g., 1-week to 6-month interventions), and/or assessment protocols. Limited evidence suggests that the intervention effects are greater in participants who are 'low active' to begin with. In particular, adolescents who already take ≥13,000-15,000 steps/day do not appear to respond to goal-setting or activity monitoring strategies using pedometers. The magnitude or pattern of change that can be expected from pedometer-based interventions in children and adolescents is not known at this time. The authors of that review concluded that since there were so few intervention studies published, yet the results were generally positive, continued research should be encouraged to inform guidelines with regards to using pedometers to promote physical activity in children and adolescents. It is clear that this area of knowledge is lacking, especially when compared with what is known about pedometer-based interventions in adults [27-29].

### Controlled studies

Cadence is the expression of steps taken per unit time (i.e., steps/minute) and it can be used to infer intensity of continuous ambulation [30,31]. Four controlled studies have been conducted with healthy young people [32-35]. The series of studies conducted by Scruggs and colleagues [36-41] were not considered here since they focus on steps detected specifically during physical education classes, which would logically include at least some sedentary time (e.g., for instruction, class management, etc.), and this would effectively lower mean cadence values. In a similar manner, a study by Beets et al. [42] focused on steps associated with time in MVPA detected during afterschool programs was not considered here.

Jago et al. [35] studied pedometer-determined steps taken by 78 11-15 year old USA-based Boy Scouts at externally-paced slow (10 minutes at 4.83 km/hr ≅ 3 METs or moderate intensity) and fast walks (10 minutes at 6.44 km/hr ≅ 5.0 METs or moderate-vigorous intensity) and running (5 minutes at 8 km/hr ≅ 8 METs or vigorous intensity) on a 200 m track. METs (metabolic equivalents) are often used to quantify physical activity intensity with respect to resting or basal metabolic rate (1 MET ≅ 3.5 ml O_2_/kg/min or 1 kcal/kg/min for adults). In the Jago et al. [35] study MET level was not directly measured but rather was inferred from the Compendium of Physical Activities [43]. Although participants also wore a CSA accelerometer (an earlier version of the ActiGraph accelerometers) during these trials, the output of that instrument was only used to assess pedometer (New Lifestyles Digiwalker SW-200) validity by correlation and was not otherwise used to inform "how many steps are enough?" Mean steps/minute overall for the slow and fast walks and the run were 117, 127, and 163, respectively. The authors focused on the results of the fast walk (taken at 5 METs) to extrapolate that approximately 4,000 steps in 30 minutes or 8,000 steps in 60 minutes was equivalent to adolescent-appropriate amounts of time in MVPA. However, if 3 METs is considered the floor of moderate intensity activity [44], it follows that 3,510 steps in 30 minutes or 7,020 steps in 60 minutes would be a more literal translation of the results of the slow 3 MET walk. It must be noted, that moderate intensity might be more correctly considered to be 4 METs in children [45]. Since cadences were only measured for 3 MET (slow) and 5 MET (fast) walks, 122 steps/min is a mid-way estimate for a 4 MET walk. This produces an estimate of 3,660 steps in 30 minutes and 7,320 steps in 60 minutes. Since Jago et al. [35] also reported that adolescents at risk of overweight (BMI > 85^th ^percentile) took somewhat fewer steps/minute (i.e., 111, 123, and 156 steps/min for each of the trials), 111 steps/min is the cadence associated with 3 METS and 117 steps/min would be the cadence associated with 4 METs. Together, the floor of moderate intensity might be better captured by a range of approximately 3,300-3,500 steps in 30 minutes (or 6,600-7,000 steps in 60 minutes) of continuous walking at 3 METs or approximately 3,500-3,700 steps in 30 minutes (or 7,000-7,400 steps in 60 minutes) at 4 METs.

Graser et al. [33] asked 34 girls and 43 boys aged 10-12 years to wear a pedometer and walk on a treadmill at 3, 3.5, and 4 miles/hour. Intensity was not directly measured; however, the authors considered these speeds to represent a range of MVPA walking intensities. The boys' and girls' cadence values were similar across the walking speeds and the researchers concluded that, in general, 120-140 steps/minute represented a reasonable cadence range associated with MVPA. Intensity-related translations based on taking 120 steps/minute at 3 miles/hour correspond to 3,600 steps in 30 minutes, or 7,200 steps in 60 minutes. Graser et al. [33] studied a somewhat younger age group than the Jago et al. [35] study and this might have produced relatively higher cadence ranges. Taken together, the two studies indicate that continuous MVPA walking (assuming at least 3 METs) produces 3,300-3,600 steps in 30 minutes or 6,600-7,200 steps in 60 minutes in 10 - 15 year olds. It is important to emphasize that such a translation should only be applied to continuous ambulation performed over the specified amounts of time. It is most important to emphasize that definitions of MVPA differed between these two studies and neither used a direct measure of intensity.

Lubans et al. [34] studied 47 boys and 59 girls (all 14 years old) walking and running on a treadmill at 65-75% of maximum heart rate (confirmed by heart rate monitor). Twenty-seven participants repeated the test three times over the course of a month to determine reliability of results. The results were highly repeatable (ICC = .83-.87). Pedometer-determined cadence associated with the designated heart rate range was 147 steps/minute (range 125 to 149 steps/minute) for boys and 137 steps/minute (range 125 to 149 steps/minute) for girls. Cadence also differed by fitness level (assessed by the 3-min Queen's College Step Test): adolescents in the lowest quintile of cardiorespiratory fitness took 129 steps/min, those in the next two quintiles averaged 138 steps/min, and those in the top two quintiles averaged 152 steps/min. It is difficult to use these cadence values to extrapolate to MVPA. The authors did not report when running vs. walking occurred, but it seems likely that the boys and girls with the top fitness levels were running at this higher cadence. Extrapolating from the adult data where the floor value (in absolute terms) of moderate and vigorous intensity is 100 and 130 steps/minute respectively [30], we would expect that a child/adolescent-specific vigorous intensity cadence is likewise at least 30 steps/minute (and likely even higher in children) more than the child/adolescent-specific moderate intensity cadence, or approximately 141 to 157 steps/minute. The Lubans et al. [34] study is grounded by a relative (vs. absolute) indicator of intensity (i.e., heart rate). Further, the heart rate range tested in this study is somewhat narrower than previously included in physical activity recommendations (i.e., 55-90% of maximum heart rate) [46]. Public health guidelines issued by the American College of Sports Medicine and American Heart Association in 2007 do not provide explicit guidelines in terms of heart rate-determined intensity [47].

More recently, Graser et al. [32] conducted another study of pedometer-determined cadence and heart-rate determined intensity in 12-14 year old adolescents. Treadmill speeds were set at 4.0, 4.8, 5.64, and 6.42 km/hr after confirming that this age group could perform all speeds without breaking into a run. These researchers defined moderate intensity as 40-59% of maximum heart rate, which may be considered low compared with physical activity recommendations (i.e., 55-90% of maximum heart rate) [46]. The corresponding cadence averaged 122 (range 108-134) steps/minute in boys and 102 (range 80-123) steps/minute in girls, suggesting great individual variation in intensity-associated cadence, a phenomenon that may reflect underlying variation in development as well as fitness. Limitations include the use of heart rate to define moderate intensity and the use of a target heart rate formula originally produced for adults. Heart rate reflects relative intensity, unlike direct measures of intensity such as MET values. As in each of the controlled studies in children and adolescents described above, steps were detected by a body-worn instrument instead of by direct observation, which is arguably the more appropriate criterion for these types of lab-based studies.

In summary, no controlled studies of cadence have used a direct measure of absolutely-defined intensity at this time and none have counted steps taken using direct observation. The limited evidence at this time suggests that, in 10-15 year olds, continuous MVPA walking produces 3,300-3,500 steps in 30 minutes or 6,600-7,000 steps in 60 minutes (assuming at least 3 METs). No studies were located that have attempted to intervene specifically on cadence. Hypothetically, however, such a practical approach might be useful for increasing time spent in MVPA.

### Translating existing physical activity guidelines

As stated earlier, public health physical activity guidelines are typically expressed in terms of frequency, time, and intensity. For example, a recent PHAC-commissioned systematic review [48] of physical activity and health concluded that "Children and youth 5-17 years of age should accumulate an average of at least 60 minutes per day and up to several hours of at least moderate intensity physical activity. Some of the health benefits can be achieved through an average of 30 minutes per day." It remains logically implicit (although not expressly stated) that these recommended minutes of at least moderate intensity be accumulated *over and above *such functional activities of daily life. There are no data at this time to inform a quantity of steps suggestive of these 'background' activities in children or adolescents, necessary to compute an estimate of steps/day that will also include recommended amounts of time spent in MVPA. However, studies of free-living behaviour present an opportunity to identify what total volume of steps/day also includes recommended amounts of activity that is of at least moderate intensity. Seven free-living studies were located that have attempted to provide such information. These studies are presented in Table 2 by year of publication. Two have focused on preschool samples [49,50], three with elementary/primary school children [15,16,51], one with adolescents recruited through primary care providers [52], and one of children and adolescents spanning 9-16 years of age recruited as part of a national survey [53].

**Table 2:** Studies of steps/day associated with time in MVPA in young people

Reference	Sample Characteristics	Instruments	Monitoring Frame	Analytical Strategy	Summary Findings
Cardon [15] 2004 Belgium	51 boys, 41 girls; elementary school children; 6.5-12.7 years	Steps/day: Yamax Digiwalker SW-200, Yamax Corp, Japan MVPA: self-report questionnaire	7 days	Linear regression equation to predict steps/day from self-reported 60 minutes in MVPA	Total: 13,130 steps/day = 60 minutes MVPA Boys: 15,340 steps/day = 60 minutes MVPA Girls: 11,317 steps/day = 60 minutes MVPA

Rowlands [16] 2005 UK	13 boys, 13 girls; primary school children; 8,3 to 10.8 years	Steps/day: Yamax Digi-Walker DW-200, Yamasa, Tokyo, Japan MVPA: Tritrac-R3D, models T303 and T303A, Professional Products, Reining International, Madison, WI	5 weekdays and 1 weekend day	Sensitivity/specificity analysis of various thresholds to ascertain likelihood of attaining 60 minutes of accelerometer-determined MVPA	Boys: 18,000 steps/day = 60 minutes MVPA Girls: 15,000 steps/day = 60 minutes MVPA

Beighle [51] 2006 USA	256 boys, 334 girls; elementary school children; 9.2 ± 1.8 years	Steps/day and Activity Time*: MLS 2505, Walk4Life, Ind., Plainfield, IL	4 weekdays	Linear regression to predict pedometer-determined daily activity time from daily step count	5,000 steps/day = 64.5 minutes of activity 10,000 steps/day = 114.5 minutes of activity 12,000 steps/day = 134.5 minutes of activity 15,000 steps/day = 164.5 minutes of activity

Cardon [49] 2007 Belgium	37 boys, 39 girls; preschool children; 4 to 5.9 years	Steps/Day: Yamax Digiwalker SW-200, Yamax Corp., Japan MVPA: MTI Actigraph, 7164 (Fort Walton Beach, FL)	2 weekdays and 2 weekend days	Regression equation to predict steps/day from accelerometer-determined time (60 minutes) in MVPA	13,874 steps/day = 60 minutes MVPA

Adams [52] 2009 USA	12 boys, 28 girls; Overweight adolescents recruited through primary care providers; 11 to 16 years	Steps and MVPA: Actigraph 7164	7 days	ROC to determine steps/day related to achieving 60 minutes accelerometer-determined MVPA; two definitions of moderate intensity used	MVPA defined at least 3 METs: 9,930 steps/day = 60 minutes MVPA MVPA defined at least 4 METs: 11,714 steps/day = 60 minutes MVPA

Tanaka [50] 2009 Japan	127 boys, 85 girls; kindergarten/nursery school children; 4.5 to 6.8 years	Steps/day: Lifecorder EX, Suzuken, Nagoya MVPA: ActivTracer, GMS, Tokyo	4 weekdays and 2 weekend days	Linear regression to predict steps/day from triaxial accelerometer-determined time (60, 100, and 120 minutes) in MVPA	9,934 steps/day = 60 minutes MVPA 12,893 steps/day = 100 minutes MVPA 14,373 steps/day = 120 minutes MVPA

Olds et al.[53] 2010 Australia	129 boys, 168 girls; 9 to 16 years	New Lifestyles 1000	randomly chosen day of six days	Linear regression equation to predict self-reported MVPA from steps/day	1 minute MVPA = 103 steps; by extrapolation 60 minutes would approximately equal 6180 steps (taken over and above lifestyle activities)

*Activity Time is not necessarily time in MVPA. Activity Time detected by this instrument is accumulated seconds of movement while the step counting lever arm is in motion.

Cardon et al. [49] reported that 13,874 pedometer-determined steps/day equated to a total volume of physical activity that included at least 60 minutes of accelerometer-determined time in MVPA in Belgian preschool children; only 8% of their sample actually achieved this level of steps/day. Tanaka and Tanaka [50] used a similar analytical approach, but collected accelerometer data using a triaxial accelerometer to conclude that 60, 100, and 120 minutes of MVPA corresponded to 9,934, 12,893, and 14,373 steps/day, respectively, in Japanese preschool children. Furthermore, 92.4%, 51.6%, and 27.4% of the sample achieved these levels. Although a direct comparison between the Belgian and Japanese studies must be tempered by the fact that different instruments were used to collect step and MVPA data, the latter sample appears to have been much more active than the former; approximately 52% of the Japanese children achieved almost 13,000 steps/day and 100 minutes in MVPA while only 8% of the Belgian sample achieved a similar value of steps/day and only 60 minutes in MVPA.

In a separate study, Cardon et al. [15] examined the relationship between 60 minutes of self-reported time in MVPA and pedometer-determined steps/day in Belgian elementary school children. Overall, 13,130 steps/day was equivalent to a total volume of daily physical activity that included 60 minutes of self-reported time in MVPA. Sex-specific values were 15,340 steps/day (boys) and 11,317 steps/day (girls). These results must be interpreted with caution; the correlation between pedometer-determined steps/day and self-reported time in MVPA was *r*=.39. In a another study comparing pedometer data with self-reported time in MVPA conducted with 9-16 year olds, the correlations ranged from .44 to .50 [53]. Linear regression was used to determine that approximately 100 steps equated to about 1 minute of MVPA. By extrapolation, the authors suggested that at least 6,000 steps would be required to accumulate 60 min of MVPA (assumedly taken over and above lifestyle activities).

Rowlands and Eston [16] conducted a sensitivity/specificity analysis of various thresholds to ascertain likelihood of attaining 60 minutes of triaxial accelerometer-determined MVPA in Welsh primary school children. They concluded that 13,000 steps/day (boys) and 12,000 steps/day (girls) provided the most reasonable estimation of attainment of 60 minutes of MVPA by way of accumulating a total volume of daily steps. Beighle and Pangrazi [51] used a pedometer that had both a step counting function and an internal stopwatch that accumulates seconds of movement while the step counting lever arm is in motion. The resulting output is labeled "activity time" but also logically includes movement that is likely performed at less than MVPA. Although the outputs were dependent (obtained from the same counting mechanism), the researchers used regression to predict daily activity time from steps/day. They reported that 5,000 steps/day was equivalent to 64.5 minutes of activity, 10,000 steps/day equals 114.5 minutes, 12,000 steps/day equals 134.5 minutes, and 15,000 steps/day equals 164.5 minutes. This study must be interpreted with due caution (and cannot be reasonably considered together with the other two studies of primary/elementary school children) since the activity time output from this instrument does not necessarily reflect time spent specifically in MVPA, but rather accumulated time associated with all detected movement.

Only a single study has attempted to translate time- and intensity-based physical activity guidelines into a steps/day value specific to adolescents [52], and this was specifically done in overweight 11-16 year olds recruited through their primary care providers. The authors used receiver operating characteristic (ROC) curves to determine a total volume of steps/day most likely related to also achieving 60 minutes of accelerometer-determined MVPA. Two definitions of moderate intensity were used (3 and 4 METs). Depending on the definition, between 10,000 (3 METs) and 11,700 (4 METs) steps/day produced the best sensitivity and specificity values for achieving at least 60 minutes of MVPA accumulated within the course of daily living.

In summary, the use of different approaches to measure steps and also time in MVPA hamper the ability to combine results and inform "how many steps are enough" in terms of attainment of recommended amounts of MVPA. Overall, limited evidence suggests that a total daily physical activity volume of 10,000-14,000 steps/day is associated with 60-100 minutes in MVPA for preschool children (≅4-6 years of age) [49,50]. Sixty minutes of MVPA in primary/elementary school children appears to be achieved, on average, within a total volume of 13,000 to 15,000 steps/day in boys and 11,000 to 12,000 steps/day in girls, although these ranges reflect findings based on both self-report [15] and triaxial-determined time in MVPA [16]. For adolescents, 10,000 to 11,700 steps/day may be associated with 60 minutes of MVPA, however there is only a single study, and it is based primarily on overweight adolescent girls [52].

### Health outcome-related analyses

Besides a translation of time in intensity, steps/day recommendations could also be informed by studies that relate step-defined physical activity to desired health outcomes. Four studies (Table 3) were located that examined steps/day related to indicators of healthy vs. unhealthy body composition in young people. Tudor-Locke et al. [23] combined pedometer data collected in 6-12 year olds from three countries (Australia, Sweden, USA) and used a contrasting groups method to identify criterion-referenced steps/day cut points related to BMI-defined normal weight vs. overweight/obese. The median value for 6-12 year olds was 15,000 steps/day for boys and 12,000 steps/day for girls. Duncan et al. [54] performed a similar analysis but using percent body fat obtained through bioelectric impedance in 5-12 year old New Zealanders. Overweight was defined as > 85^th ^percentile and compared with nonoverweight (< 85^th ^percentile). The authors reported that 16,000 steps/day (boys) and 13,000 steps/day (girls) were the best predictors of body fat percent-defined weight status. Laurson et al. [55] used ROC analysis to match sensitivity and specificity of various cut points and to identify the optimized cut point (which minimized misclassification error for normal weight vs overweight/obese children) in a sample of U.S. children. The optimized cut points approximated 13,500 steps/day (boys) and 10,000 steps/day (girls). Dollman et al. [56] also used ROC analysis in a sample of 2,071 5-16 year old Australian children. The optimized cut points for discriminating between normal weight and overweight/obese children were 12,000 steps/day for 5-12 year old boys, 10,000 steps/day for 5-12 year old girls, and 11,000 steps/day for 13-16 year old boys. The optimized cut point for 13-16 year old girls (14,000 steps) did not significantly discriminate between those who were classified as normal weight versus overweight.

**Table 3:** Studies of steps/day related to indicators of healthy vs. unhealthy body composition in young people

Reference	Sample Characteristics	Step Counting Instrument	Monitoring Frame	Analytical Strategy	Findings
Tudor-Locke [23] 2004 Australia Sweden USA	959 boys, 995 girls; school children; 6 to 12 years	MyLife Stepper MLS-2000 Yamax, Tokyo, Japan	4 week days	Contrasting groups method to identify optimal steps/day related to BMI- defined normal weight vs. overweight/obese IOTF	Boys: 15,000 steps/day Girls: 12,000 steps/day

Duncan [54] 2006 New Zealand	454boys, 515girls; elementary school children; 5 to 12 years	NL-2000, New Lifestyles Inc., Lee's Summit, MO	3 weekdays, 2 weekend days	Contrasting groups method to identify overweight vs. nonoverweight based on 95^th ^percentile for % body fat by bioelectric impedance	Boys: 16,000 steps/day Girls: 13,000 steps/day

Laurson [55] 2008 USA	358 boys, 454 girls; elementary school children; 6 to 12 years	Digiwalker 200-SW	4-7 days including at least one weekend day	ROC, specificity, sensitivity to determine maximal accuracy of identifying BMI-defined normal weight vs. overweight/obese (IOTF criteria)	Boys: 13,666 Girls: 9,983

Dollman [56] 2010 Australia	995 boys, 1,076 girls; Nationally representative sample; 5 to 16 years	New Lifestyles 1000	7 days including weekends	ROC, specificity, sensitivity to determine maximal accuracy of identifying BMI-defined normal weight vs. overweight/obese (IOTF criteria)	Boys (5-12 years): 12,000 Boys (13-16 years): 11,000 Girls (5-12 years): 10,000 Girls (13-16 years): NS findings

Note: IOTF = International Obesity Task Force [58]

In summary, the two studies that have applied the contrasting groups method applied to different weight status criteria (BMI and percent body fat) have produced consistently high values for steps/day: 15,000-16,000 steps/day for boys and 12,000-13,000 steps/day for girls [23,54], but these findings may be an artefact of the samples studied [24]. The ROC analyses conducted in the other two studies [55,56] demonstrate better sensitivity and specificity with much lower values of steps/day (approximately 10,000-13,500 steps/day). Although Eisenmann et al. [57] reported that children not meeting the BMI-reference cut points were more likely to be classified as overweight, Beets et al. [24] also reported concerns about the sensitivity and specificity of the BMI-referenced cut points, and in particular questioned their utility across countries, for example in the U.S. where activity levels are lower (i.e., where even normal weight children have relatively lower activity levels). Across studies, the lowest estimate has been 10,000 steps/day, and most can agree that even lower values are of increasing concern, and higher values are increasingly desirable. However, since BMI is obviously influenced by more than just ambulatory activity, it may be more appropriate to seek agreement on a step-based translation of public health guidelines than to pursue a more precise estimate associated with a healthy BMI in children and adolescents that is also universally applicable at this time.

## Discussion

Drawing from the studies reviewed above, the minimal recommendation (embodied in most public health guidelines world-wide) of 60 minutes of MVPA is associated with 10,000-14,000 free-living steps/day in preschool children (≅4-6 years of age), 13,000 to 15,000 steps/day in male primary/elementary schoolchildren, 11,000 to 12,000 steps/day in female primary/elementary school children, and 10,000-11,700 steps/day for adolescents. Boys and girls appear to be more similar in their step patterns during the preschool years and again in the adolescent years. In contrast, the consistent sex-associated discrepancy in steps/day observed in primary/elementary schoolchildren, perhaps most clearly illustrated in the sex-and-age specific graphs assembled by Beets et al.[12] representing data from 13 different countries, and the differential empirical evidence related to step-defined attainment of public health guidelines, is difficult to ignore but must continue to be debated and evaluated.

Overall, the primary/elementary schoolchildren values are reasonably compatible with matched normative data [11,12] and fit within ranges of criterion-referenced data that have been associated with healthy body composition parameters [23]. Since adolescents (compared to children) steadily decline in their daily physical activity levels [11,12], the step/based recommendations above (e.g., translations of time- and intensity-based guidelines) are higher than existing normative data and therefore will be more difficult (but neither necessarily impossible nor contraindicated) for adolescents to achieve. There are no step values based on any health parameters (e.g., BMI, body fat percent, blood pressure, etc.) for adolescents or preschool children at this time to aid in interpretation.

**Figure 1:**
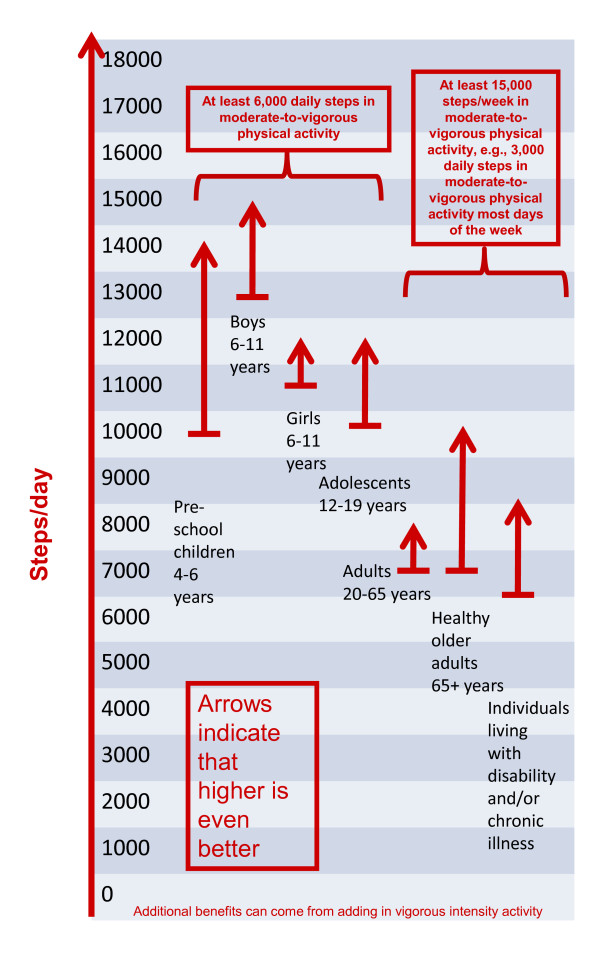
**Steps/day scale schematic linked to time spent in MVPA**.

As noted above, the notion of a graduated step index has been introduced for children [21]. The anchors for this index have been criticized [24] and there remains a concern about the appropriateness of labeling children as 'sedentary' [21]. An improvement to the original graduated step index would be to offer a more fully expanded steps/day scale. Such a scale would incorporate child and adolescent-specific step-based translations of public health guidelines within the context of the full lifespan, but also provide additional incremental "rungs" corresponding with roughly 10-minute bouts of activity. It would begin at zero and continue to 18,000+ steps/day (the highest mean value reported for any sample at this time, that is, Amish men [19]). Adoption of such a scale would be useful for both research and health promotion purposes. A schematic of this concept showing 1,000 step increments is presented in the accompanying Figure 1. The ranges shown in Figure 1 represent the best evidence (albeit preliminary) linking objectively monitored time in MVPA with steps/day.

The implied relationship between steps/day and various health outcomes is a primary consideration for setting any steps-based translation of physical activity guidelines. However, the data patterns presented above suggest that step cut points should also consider natural variation in average activity levels associated with age. However, then the concern is that as populations experience anticipated societal decreases in average activity levels, normative-based cut points would also decrease over time. This would make any static, standardized cut point out of touch with local reality, and therefore less useful. Standardized cut points, however they are set, would facilitate global comparisons (and they could be used to illuminate gross discrepancies in between-country behaviour). The compromise is within-country cut points that reflect normative behaviour and also include locally-relevant incremental levels, thereby providing additional "rungs on the ladder" for promotion of activity in less active populations. However, such a strategy must be cautiously applied to avoid validating or otherwise absolving, or even endorsing, underachievement and thus promoting perpetuation of a low active lifestyle.

## Conclusions

The evidence accumulated to date indicates that there is no simple or "magical" number of steps/day that cuts across all ages. Preschool children are different from primary/elementary school age children, and children are different from adolescents, and the objectively monitored data presented in this review support this. In a similar way, dietary guidelines have historically recommended different amounts of various food groups depending on sex and age. Applying the findings reviewed herein, primary/elementary schoolchildren would be directed (in both public health messages and targeted interventions) to higher levels (boys 13,000-15,000 steps/day; girls 11,000-12,000 steps/day, as indicated by collected evidence reviewed above), adolescents (10,000-11,700 steps/day) would be intermediate to children and adults [8], and adults and older adults [9] directed to the ranges more specifically appropriate for them, adjusting of course in consideration of abilities and lifestyles that must accommodate disability or chronic illnesses. No potentially stigmatizing labelling would be applied. Regardless, however, every individual would be able to identify their level and the ones immediately above.

If adopted, such a steps/day scale should continue to reinforce the importance and added value of taking at least an age-appropriate portion of daily steps (e.g., 6,000 steps over 60 minutes) at minimally moderate intensity, and if at all possible, at vigorous intensity, congruent with public health guidelines world-wide. Of course, non-ambulatory moderate and vigorous intensity activities (e.g., swimming, bicycling) are also valuable. Recommendations are based on a limited number of relevant studies and must therefore be considered preliminary. Further research is needed to confirm and extend values for directly measured cadences, associated speeds, and MET values in young people; continue to accumulate normative data (expected values) for both steps/day and MVPA across ages and populations; and, conduct more cross-sectional, longitudinal, and intervention studies in children and adolescents to inform the shape of dose-response curves of step-defined physical activity associated with various health parameters.

## Competing interests

The following authors declare they have no competing interests: CT-L, MWB, SB, GMC, SD, YH, DRL, TSO, AR, DAR, JCS, and ST. CLC is associated with the Canadian Fitness and Lifestyle Research Institute which is funding in part by the Public Health Agency of Canada (PHAC). AR has served as medical advisor for Keep Walking Scandinavia AB, a company in the wellness sector with online consulting, online distribution of literature and online distribution of pedometers of different brands. SNB receives book royalties (<$5,000/year) from Human Kinetics; honoraria for service on the Scientific/Medical Advisory Boards for Alere, Technogym, Santech, and Jenny Craig; and honoraria for lectures and consultations from scientific, educational, and lay groups. During the past 5-year period SNB has received research grants from the National Institutes of Health, Department of Defence, Body Media, and Coca Cola.

## Authors' contributions

CT-L and CLC conceived and designed the project. CT-L acquired the data and prepared analysis for initial interpretation. All authors contributed to subsequent analysis and interpretation of data. CT-L prepared a draft of the manuscript. All authors contributed to critically revising the manuscript for important intellectual content. MWB, JSD, DRL, TSO, edit checked the tables. All authors gave final approval of the version to be published and take public responsibility for its content.
